# Adenosine A1R/A3R agonist AST-004 reduces brain infarction in mouse and rat models of acute ischemic stroke

**DOI:** 10.3389/fstro.2022.1010928

**Published:** 2022-11-22

**Authors:** Elizabeth S. Fisher, Yanan Chen, Mikaela M. Sifuentes, Jeremy J. Stubblefield, Damian Lozano, Deborah M. Holstein, JingMei Ren, Matthew Davenport, Nicholas DeRosa, Tsung-pei Chen, Gerard Nickel, Theodore E. Liston, James D. Lechleiter

**Affiliations:** 1 Department of Cell Systems and Anatomy, University of Texas Health at San Antonio, San Antonio, TX, United States; 2 NeuroVasc Preclinical Services, Inc., Lexington, MA, United States; 3 Astrocyte Pharmaceuticals Inc., Cambridge, MA, United States

**Keywords:** *Adora3*, stroke treatment, mitochondrial metabolism, ATP, astrocytes

## Abstract

Acute ischemic stroke (AIS) is the second leading cause of death globally. No Food and Drug Administration (FDA) approved therapies exist that target cerebroprotection following stroke. Our group recently reported significant cerebroprotection with the adenosine A1/A3 receptor agonist, AST-004, in a transient stroke model in non-human primates (NHP) and in a preclinical mouse model of traumatic brain injury (TBI). However, the specific receptor pathway activated was only inferred based on *in vitro* binding studies. The current study investigated the underlying mechanism of AST-004 cerebroprotection in two independent models of AIS: permanent photothrombotic stroke in mice and transient middle cerebral artery occlusion (MCAO) in rats. AST-004 treatments across a range of doses were cerebroprotective and efficacy could be blocked by A3R antagonism, indicating a mechanism of action that does not require A1R agonism. The high affinity A3R agonist MRS5698 was also cerebroprotective following stroke, but not the A3R agonist Cl-IB-MECA under our experimental conditions. AST-004 efficacy was blocked by the astrocyte specific mitochondrial toxin fluoroacetate, confirming an underlying mechanism of cerebroprotection that was dependent on astrocyte mitochondrial metabolism. An increase in A3R mRNA levels following stroke suggested an intrinsic cerebroprotective response that was mediated by A3R signaling. Together, these studies confirm that certain A3R agonists, such as AST-004, may be exciting new therapeutic avenues to develop for AIS.

## Introduction

Acute ischemic stroke (AIS) occurs in ~795,000 individuals annually in the US, resulting in over 147,000 deaths, and often permanent disability in those who survive (Virani et al., 2021). Globally this number rises to over 6.5 million annual deaths (GBD 2019 Stroke Collaborators, 2021). Current approved treatments are limited and focus only on restoration of cerebral blood flow to the ischemic area of the brain, achieved by either intravenous administration of tissue plasminogen factor (tPA) to dissolve blood clots and/or endovascular mechanical thrombectomy to remove a large vessel blood clot causing ischemia (National Institute of Neurological Disorders Stroke rt-PA Stroke Study Group, 1995). These therapies are constrained to small subsets of patients in the emergency departments of both primary and comprehensive stroke centers; ranging from 10 to 14% of patients for tPA and from 1 to 4% of patients for endovascular clot removal, respectively (Man et al., 2018). Moreover, neither therapy directly targets neuronal survival within the ischemic penumbra, which if rescued would lead to better patient outcomes (Meyers et al., 2011; Powers et al., 2015).

Loss of oxygen and glucose during ischemia dysregulates many energy-dependent processes in the brain, leaving affected tissue at significant risk of damage and cell death (Parpura et al.,2017). While brain tissue at the core of an infarction is rapidly and irreversibly lost, cells in the surrounding hypoperfused penumbra die over hours and days after the initial ischemic event, and thus have the potential to be rescued (Tymianski, 2013; Grupke et al., 2015). Research by our group demonstrated the intrinsic healing mechanisms of astrocytes could be enhanced by improving their mitochondrial ATP production, leading to an energy-dependent reduction in the size of brain lesions in mouse models of stroke (Zheng et al., 2010, 2013). This early research focused on P2Y_1_ receptor activation (P2Y_1_R), using the P2Y_1/12/13_ receptor agonist 2-methylthio-adensoine di-phosphate (2-MeSADP) and then the specific P2Y_1_R agonist MRS2365. Subsequent research on the pharmacokinetics (PK) and metabolism of these compounds indicated MRS2365 was a prodrug that was rapidly and completely metabolized *in vivo*, to the novel nucleoside metabolite AST-004 (Liston et al., 2020). Cerebroprotection with these first-generation purinergic agonists (Zheng et al., 2010, 2013; Talley Watts et al., 2013) was attributed to the conformationally constrained metabolite of MRS2365, AST-004, and the non-conformationally constrained metabolite for 2MeS-ADP, 2-methylthioadenosine (Liston et al., 2020).

We recently confirmed the cerebroprotective efficacy of AST-004 treatments in a preclinical mouse model of traumatic brain injury (TBI) and a transient stroke model in non-human primates (NHP) (Liston et al., 2022). However, the specific receptor pathway activated was only inferred from binding studies showing AST-004 interacted with the adenosine A3 receptor (A3R), with some affinity for the adenosine A1 receptor (A1R) (Liston et al., 2020). Here, we further investigated the underlying mechanism of AST-004 cerebroprotection in two independent models of AIS: permanent photothrombosis stroke in mice and transient middle cerebral artery occlusion (MCAO) in rats. Our working hypothesis was that AST-004 cerebroprotection was mediated by A3R agonism and was dependent on stimulation of astrocyte mitochondrial metabolism.

We found AST-004 treatments were effective across a range of doses and that cerebroprotection could be blocked by the A3R antagonist MRS1523. The high affinity A3R agonist MRS5698 also decreased infarct size in the mouse photothrombotic model, whereas the high affinity A3R agonist Cl-IB-MECA was ineffective under our mouse photothrombosis experimental conditions. Cerebroprotection was blocked by the astrocyte specific mitochondrial toxin fluoroacetate, indicating a mechanism of action dependent on astrocyte mitochondrial ATP production. We also found increased levels of A3R mRNA following stroke, revealing an intrinsic protective response that could be exploited for treatment. A3R agonists have been reported as cerebroprotective against stroke since the mid-1990s (Rudolphi et al., 1992; von Lubitz et al., 1994, 1995, 1999, 2001; Rudolphi and Schubert, 1995; Fedorova et al., 2003; Choi et al., 2005, 2011; Chen et al., 2006; Bjorklund et al., 2008), and most recently for TBI (Farr et al., 2020; Bozdemir et al., 2021). Together, these studies confirm that certain A3R agonists, such as AST-004, may be exciting new treatment options that need to be developed for clinical evaluation.

## Materials and methods

### Animals

Male and female C57/BL6 mice with access to food and water *ad libitum* were housed in 12 h light-dark cycles. Experimenter was blinded to experimental groups. All mouse experiments were performed on mice aged between 3 and 6 months, in accordance with the Institutional Animal Care and Use Committee at UT Health San Antonio. All rat surgeries were performed at NeuroVasc Preclinical Services Inc. (Lexington, MA). Preclinical services and procedures reviewed and approved by the IACUC at NeoSome (Lexington, MA). Male Wistar rats 225–250 g (8 weeks) were ordered 7–10 days prior to surgery (Charles River Laboratories, Wilmington MA). They were allowed free access to food and water and housed two per cage.

### Photothrombotic stroke

#### Method #1

All photothrombic strokes were induced using this protocol as previously described (Zheng et al., 2010, 2013), except for the data presented in Figures 5A,C. Briefly, mice were anesthetized with 4% isoflurane and maintained at 2% isoflurane throughout the surgery. Hair was removed, and incision made on the dorsal scalp, and head mounted in a custom frame. Either a cranial window or a thin skull prep was performed. Rose Bengal (Sigma, Cat no 330000) dye was then injected intravenously, and a blood clot induced with a 568 nm laser on a Nikon (TE 200) microscope, with blood vessels between 30 and 40μM targeted for clotting. Mice were injected with drugs [MRS2365: Tocris Cat no 2157; MRS1523: Sigma Cat no M1809; Fluoroacetate: Sigma Cat no 62-74-8; MRS5698: Tocris Cat no 5428; Cl-IB-MECA: Tocris Cat no 1104; AST-004, synthesized at the National Institute of Diabetes, Digestive and Kidney Diseases, Bethesda, MD (Ravi et al., 2002)] either before surgery or 30 min post-stroke, as described in the paper.

#### Method #2

For the experimental data presented in Figures 5A,C, a second photothrombotic procedure was used as recently described (Alamri et al., 2021). In brief, male C57/BL6 mice (3 months old) were anesthetized with isoflurane and hair from the top of the head was removed by chemical depilatory (Nair, over-the-counter). The mouse was placed on a surgical platform where the head was cleaned, using aseptic techniques. A 1.5 cm midline incision was then made through the skin. The connective tissue covering the skull was removed using a small scissors followed by cleaning of the skull surface with a hydrogen peroxide swab. Mice were subsequently injected with Rose Bengal solution (8 mg/mL, 10 mL/kg) by intraperitoneal administration 5 min prior to stroke. During this 5 min period, Bregma 0 was located under a dissecting microscope. A fiber optic illuminator (2.16 mm optic cable) was placed 1.7 mm lateral to midline and Bregma 0 in the right hemisphere of the mouse. The right hemisphere was illuminated for 15 min through the intact skull with a 120 mW 561 nm laser (Coherent sapphire CDRH driver unit set at 38%, 45 mW). All laser procedures were performed in a class 4 laser safety room with laser curtain. After illumination, the probe was removed and the incision closed with sutures. The sutured wound was gently cleaned by chlorhexidine and a thin layer of the first aid antibiotic pain relieving. After surgery, mice were kept in a recovery chamber (~37°C) for ~1.5 h before returning them to their cages.

### TTC staining and lesion volume quantification

2,3,5-Triphenyltetrazolium chloride (TTC, Sigma Aldrich, Cat no T8877) staining was performed as previously described. Briefly, brains were removed and placed in ice-cold PBS for 5 min on ice. Then brains were placed in a tissue matrix (Ted Pella, Cat no 15050), and sliced into 1-mm thick coronal section through the brain and placed in solution with TTC for 16 min at 37 degrees Celsius, turning over halfway through incubation. Once stained, sections were fixed overnight with 4% PFA.

### Scanner-based image acquisition

TTC stained coronal sections were imaged on an Epson V850 pro scanner (dual lens systems with high pass optics). Care was taken to ensure that sections did not come into contact with each other. Slices were sequentially placed on a transparency, ordered rostral to caudal, then a second transparency was placed on top of the slices. This permitted the slices to be turned without damaging them. A 15 cm ruler (provided by Fine Science Tools) was also placed near the top of each transparency for calibration. Once scanned, the transparencies sandwiching the slices were flipped to scan the other side. The scanner was controlled with Epson software (Ver 3.9.3.4 US) and set to professional mode, which acquires 24-bit color images at 1,200 dpi image resolution, no color correction or image enhancement, linear gain and a gamma equal to 2.2. The exposure level setting was set to medium and the densitometer sampling area setting was set at 1 × 1 pixels. The resulting image was saved as an uncompressed tagged image file (tiff) format.

### Image analysis and infarct size determinations

Analysis was performed using NIH Image J. (version 1.53K). A measurement scale setting was initiated using the line draw tool. A horizontal line was drawn from 1 to 2 cm on the ruler. Under the “Analyze” pull down menu, “Set Measurements” was selected and the distance in pixels was set to 235.0021, with a known distance of 10 and the pixel aspect ratio set at 1.0. The “Global” box was checked to permit measurement of multiple images for each series of coronal sections. The image for each coronal sections was zoomed to 300% prior to outlining the lesion area with the freehand button on the toolbar. The area of each lesion was read off the “Measure” tool, selected under the “Analyze” pull down menu. The lesion volume per slice was calculated as the average of the front and back side of each slice times 1 mm, the thickness of each section. Slice volumes were then summed to calculate the total infarct volume per mouse brain.

### Western blotting

A 1 mm punch was removed each from ipsilateral and contralateral sides of a stroke brain. The punch was then sheered in sample buffer and sonicated for 10 min and spun at 10,000 rpm. Supernatants were collected and 50 μg of protein was loaded onto a 10% SDS gel and run and transferred to a nitrocellulose membrane. Blots were then probed for GFAP (Dako Omnis Cat no Z0334) at 1:1000 and GAPDH (Cell Signaling Cat no 97166S) 1: 1000. LiCor secondary antibodies (donkey anti-rabbit Cat no 926032213 and donkey anti-mouse Cat no 926-68022) were used and images were developed using the Odyssey system.

### RNA isolation and quantitative real time-PCR

#### Tissue harvest

Twenty-four hours after stroke, mice were euthanized, and their brains were rapidly removed, and 1 mm serial coronal sections were collected. Following TTC staining and scanning, brain sections were placed under a dissection microscope and the region of tissue classified as “stroke lesion” (i.e., the section of the brain that remained white following incubation in the TTC solution) was removed through cutting with a scalpel or pair of fine dissection scissors (Fine Science, Cat no 15018-10). The stroke lesion samples were pooled in a 1.5 mL microcentrifuge tube, weighed, and then flash-frozen in liquid nitrogen. The side of the brain with a stroke lesion was termed “ipsilateral.” An equivalently sized piece of tissue was cut from the opposite side of the brain (contralateral) on each section that displayed a stroke lesion. The contralateral pieces were also pooled in a separate 1.5 mL microcentrifuge tube, weighed and flash-frozen in liquid nitrogen. Tubes were then stored in a −80°C freezer for downstream processing.

#### RNA isolation and cDNA synthesis

Frozen ipsilateral and contralateral tissue was ground into a tissue powder using a liquid nitrogen mortar and pestle. The tissue powder was transferred to a 2 mL microcentrifuge tube and 1 mL TRIzol Reagent (ThermoFisher, Cat no 15596018) was added. The tissue powder sample was homogenized for 15–20 s in TRIzol using a mechanical hand-held homogenizer. Following homogenization, samples were centrifuged at 12,000 × g for 10 min in a 4°C centrifuge and the supernatant was transferred to a separate tube for RNA isolation. The RNA isolation procedure was then followed according to the manufacturer’s instructions. Isolated RNA was resuspended in DEPC-treated ddH_2_O and checked for concentration and quality of a Nanodrop 2000. RNA samples were diluted to 200 ng/μL and 1 μg of RNA was converted into cDNA using a High-Capacity cDNA Reverse Transcription Kit according to the manufacturer’s instructions (ThermoFisher, Cat no 4368814). cDNA was then diluted 1:5 in ddH_2_O for downstream analysis.

#### qRT-PCR

Diluted cDNA (1:5 dilution) was used for quantitative real time-PCR (qRT-PCR). Genes of interest were normalized to *Gapdh* gene expression. Expression was determined using the ΔΔCT method. Primer sequences were designed using Primer-BLAST (http://www.ncbi.nlm.nih.gov/tools/primer-blast/). Primer sequences were as follows: *Gapdh* (Forward 5′-3′ CAAGGAGTAAGAAACCCTGGACC, Reverse 5′-3′ CGAGTTGGGATAGGGCCTCT) (Stubblefield et al., 2018), *Gfap* (Forward 5′-3′ AAAACCGCATCACCATTCCTG, Reverse 5′-3′ GTGACTTTTTGGCCTTCCCC), and *Adora3* (Forward 5′-3′ GACAGTCAGATATAGAACGGTTACCAC, Reverse 5′-3′ TTCCAGCCAAACATGGGGGTCA). qRT-PCR amplification of target genes was achieved using Power SYBR Green PCR Master Mix (ThermoFisher, Cat no 4368706) and primer forward/reverse mixes at a final primer concentration of 150 nM in a 10 μL reaction on a 384-well plate. qRT-PCR was conducted on an Applied Biosystem 7900HT Real-Time PCR System.

### Rat tMCAO surgeries

The middle of the neck was shaved with electric clippers and cleaned with Hibiclens. A skin incision was made over the right common carotid artery (CCA), the muscle was retracted, and the CCA bifurcation was exposed. The CCA was ligated, and a distal segment of the external carotid artery (ECA) was temporarily clamped using a suture or clip. A nylon suture was then inserted through the CCA and advanced into the internal carotid artery (ICA) for a predetermined distance (18–21 mm) based on animal weight. The ECA clip/suture was then removed. After a cannulation of the right jugular vein with PE-90 tubing, the skin incision was closed with surgical staples. The animal was again anesthetized after the 90 min ischemic period, the wound was re-opened, and the intravascular suture was removed from the CCA, initiating reperfusion. The bolus dose, followed by the primed pump connection, was administered immediately upon suture removal and the skin wound was again closed. During the time of anesthesia, a self-regulating heating pad connected to a rectal thermometer was used and maintained at 37.0° ± 1°C. Cefazolin (40 mg/kg; Hikma Parma Corp 156005) was given intraperitoneally. before surgery to prevent infections. Subcutaneous buprenorphine (~0.1 mg/kg; Par Pharm companies, Inc.) was given before surgery as analgesia. All treatment solutions were stored in 4°C and kept in 4°C until use. Vehicle: DMSO, High Dose Group 3 mg/kg bolus and 0.042 mg/min/kg Alzet Infusion, Mid Dose Group 0.4 mg/kg bolus and 0.0056 mg/min/kg Alzet Infusion, Low Dose Group 0.04 mg/kg bolus and 0.00056 mg/min/kg Alzet Infusion. Starting at the time of reperfusion, animals received 1 ml/kg intravenous bolus followed by Alzet Infusion (8 μl/h for 24 h) through jugular vein. These dosing regimens were designed to maintain targeted steady-state plasma and brain concentrations of AST-004 throughout the evaluation period. Treatments were randomly assigned to each day of surgery: (https://www.randomizer.org).

### Rat functional assay

Functional activities were evaluated using modified neurological rating scale (mNRS) (Longa et al., 1989). Modified Neurological Rating Scale (mNRS): 0—Indicated no neurologic deficit; 1—Failure to extend left forepaw fully, a mild focal neurologic deficit; 2—Circling to the left, a moderate focal neurologic deficit; 3—Falling to the left, a severe focal deficit: 4—Rats did not walk spontaneously and had a depressed level of consciousness. 5—Death.

### Rat sacrifice and lesion volume quantification

Twenty-four hours after reperfusion/dosing, rats were sacrificed using CO_2_, and brains were removed and cut into seven 2 mm thick coronal sections using a rat brain matrix (+4.7, +2.7, +0.7, −1.3, −3.3, −5.3, and −7.3, compared to bregma, respectively), and stained with TTC. The brain sections were put into 2% TTC solution in a dark place at room temperature for 30 min. The TTC solution was then changed to 10% formalin for fixation until images were captured ~24 h later. Images were captured using a digital camera fixed on a photo stand. Volumetric analysis of the infarct area was performed using Image J (NIH software). The free-hand tool was used to trace the area of the infarcted tissue of the right hemisphere, the uninfarcted tissue for both hemispheres. Infarct area was calculated by subtracting the uninfarcted area of the ipsilateral hemisphere from the area of the intact contralateral hemisphere. The volume of each hemisphere was then calculated by multiplying the area with section thickness (2 mm) and the number of sections in between each sampling (7). The infarct volume was expressed as a percentage of the intact hemispheric volume.

### Statistics

Statistical analysis was performed in GraphPad/Prism. All data were expressed as mean ± S.E.M. The significance level (alpha level) was set to 0.05 (5%). All pairwise comparisons were performed using student’s *t*-test. One way-ANOVA was used in the multiple dosing experiments, and for examining inter-sex differences. Rat data was analyzed by one way ANOVA.

## Results

### AST-004 is cerebroprotective in mice after permanent photothrombotic occlusion over a wide range of doses

**FIGURE 1:**
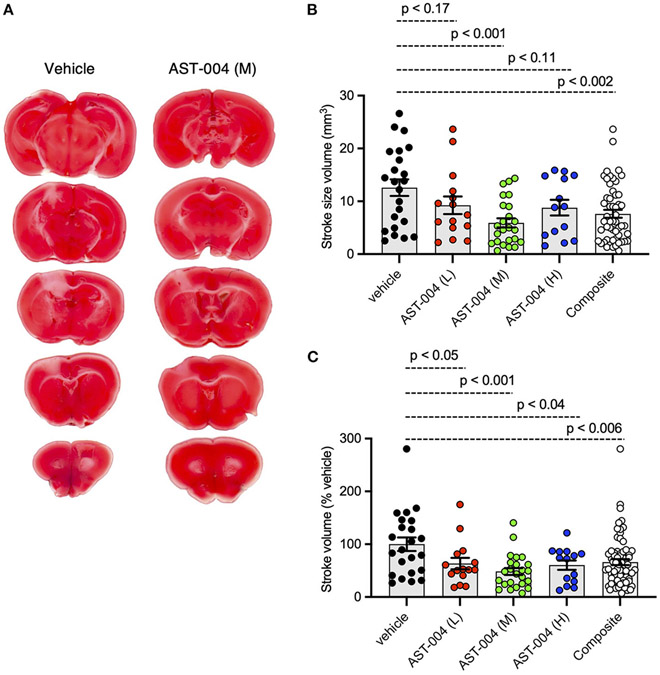
Photothrombosis-induced stroke infarctions are reduced by AST-004 treatments. **(A)** Coronal sections of brains stroked with photothrombosis for vehicle (saline injected) and AST-004 (mid dose) treated mice. **(B)** Average TTC-stained stroke volumes in mice treated with either vehicle or AST-004 at the concentrations labeled: 0.022 mg/kg (L), 0.22 mg/kg (M), and 2.2 mg/kg (H). Composite group are all values from L, M, and H AST-004 doses. **(C)** Data in **(B)** normalized to the mean vehicle lesion size and replotted. Data were pooled from three experiments using male mice and plotted as mean +/− SEM.

To test whether the A1R/A3R agonist, AST-004, was protective against cerebral ischemia, we induced stroke using photothrombosis and intraperitoneally (IP) injected AST-004 within 30 min of stroke onset. Twenty-four hours post-stroke, brains were harvested, coronally sliced into 1 mm sections, then stained with triphenyltetrazolium chloride (TTC), a dye which turns red under dehydrogenase activity in live tissue. Cortical tissue near the site of injury that remained white was considered part of the ischemic lesion. Lesion volumes were estimated by serially integrating the lesions areas in each 1 mm-thick brain section. Control mice injected with vehicle alone exhibited an average lesion volume of 12.59 +/− 1.56 mm^3^ (*n* = 23) (Figure 1A). We tested 3 AST-004 doses a full log difference from each other: low (0.022 mg/kg), mid (0.22 mg/kg), and high (2.2 mg/kg) concentrations. Previous work by our group had demonstrated significant cerebroprotective efficacy after traumatic brain injury at the mid-dose (0.22 mg/kg) (Bozdemir et al., 2021). We found that all 3 doses significantly decreased lesion volume (Figures 1B,C). The low dose of AST-004 (L) reduced lesion size to 9.26 +/− 1.67 mm^3^ or 63.38 +/− 10.87% of vehicle treated mice (*n* = 15, *p* < 0.05). Those mice treated with the middle AST-004 (M) dose following photothrombotic stroke showed significantly reduced lesion volumes with an average of 5.92 +/− 0.88 mm^3^ or 48.39 +/− 6.59% of vehicle (*n* = 24, *p* < 0.001), while the high dose of AST-004 (H) reduced the average lesion size to 8.80 +/− 1.48 mm^3^ or 60.38 +/− 8.92% of vehicle (*n* = 14, *p* < 0.04; Figures 1B,C). The composite lesion size when all 3 doses of AST-004 were pooled was 7.63 +/− 0.75 mm^3^ or 66.05 +/− 5.67% of vehicle (*n* = 53, *p* < 0.006).

### AST-004 is cerebroprotective in mice following stroke in females and males

**FIGURE 2:**
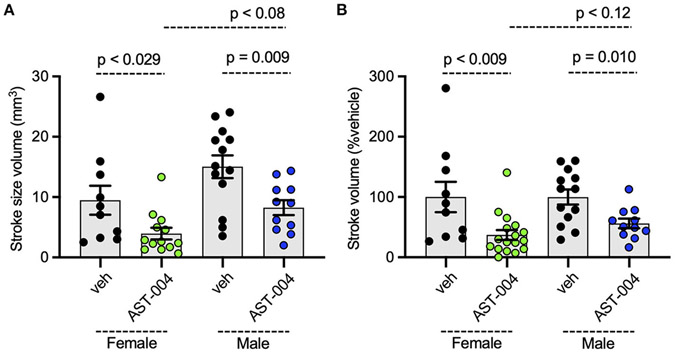
Photothrombosis-induced stroke infarctions are reduced by AST-004 in both male and female mice. **(A)** Average TTC-stained stroke volumes for male (M) and female (F) mice. **(B)** Female and male groups replotted as a % of their vehicle-treated means. Data were pooled from three experiments and plotted as mean +/− SEM.

To assess whether AST-004 efficacy was sex-dependent, we re-plotted the data presented in Figure 1, separating lesion data into groups for male and female mice. We found no significant differences between female and male average lesion sizes, 9.48 +/− 2.40 mm^3^ (*n* = 10) vs. 15.04 +/− 1.89 mm^3^ (*n* = 13), respectively, in vehicle treated mice. Lesion sizes for AST-004 treated mice were significantly reduced for females, 3.95 +/− 0.95 mm^3^ (*p* < 0.029, *n* = 13) or 37 +/− 8.18% of vehicle and also for males, 8.25 +/− 1.25 mm^3^ (*p* < 0.009, *n* = 11) or 56.20 +/− 7.91% vehicle, indicating sex was not a determinant for AST-004 efficacy (Figures 2A,B). We note that although male lesion sizes trended higher, even when expressed as a % of the mean vehicle lesion size, these differences were not statistically different (Figures 2A,B).

### AST-004 cerebroprotection is blocked by an A3R antagonist

**FIGURE 3:**
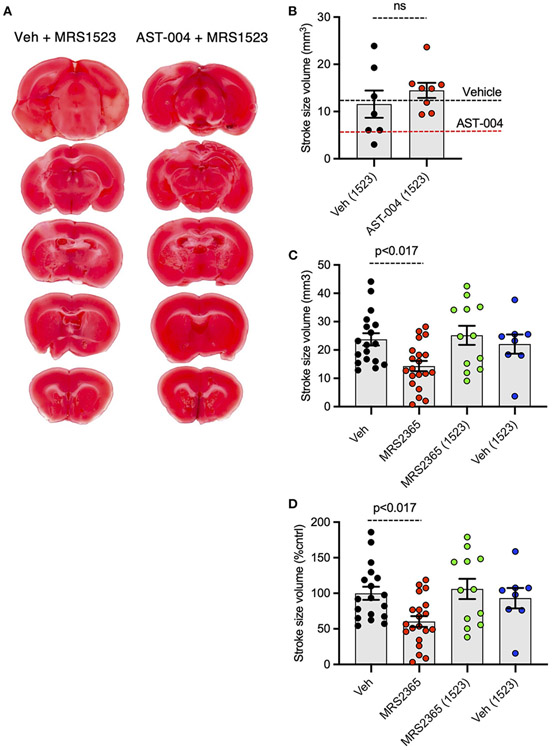
Photothrombosis-induced stroke infarctions reduced by AST-004 or by MRS2365 are blocked by A3R antagonist MRS1523. **(A)** Coronal sections from stroked mice pre-injected 15 min before stroke onset with the A3R antagonist MRS1523, stroked, and post-treated with vehicle or AST-004. **(B)** Average TTC-stained stroke volumes in mice treated with MRS1523 alone or with AST-004 and MRS1523. **(C,D)** Average TTC-stained stroke volumes in mm^3^ **(C)** or as a % of vehicle treated **(D)** in mice treated with MRS2365 with or without MRS1523.

We recently reported AST-004 was primarily a moderate affinity A3R (human K_i_ 1490 nM) and A1R (human K_i_ 1590 nM) agonist (Liston et al., 2020). For comparison, the natural endogenous ligand, adenosine, has K_*i*_ values of 70 and 6,500 nM for human A1Rs and A3Rs, respectively (Dunwiddie and Masino, 2001). This equates to ~4x higher affinity of AST-004 for human A3Rs and to ~23× lower affinity of AST-004 for human A1Rs compared to adenosine. Accordingly, we tested whether cerebroprotection was inhibited by pre-injecting mice with the A3R antagonist, MRS1523 (2 mg/kg), 15 min before inducing a photothrombotic stroke. MRS1523 (2 mg/kg) was also added along with vehicle or AST-004 after each stroke. Brains were harvested 24 h post stroke, sliced, stained for TTC, and lesion volumes measured as described in Figure 1. We found that MRS1523 by itself, did not significantly affect the average lesion volume (Figures 3A,B). The mean lesion size was 11.57 +/− 2.90 mm^3^ (*n* = 7), comparable to the average lesion size for mice treated with only vehicle (dashed line, Figure 3B). In contrast, MRS1523 completely blocked AST-004 efficacy. The average lesion size was 14.48 +/− 1.60 mm^3^ (*n* = 8, pooled from low and high AST-004 treated mice), not significantly different than the average lesion size in control mice. For comparison, the average lesion size in mice treated with AST-004 alone is shown as the dashed red line (Figure 3B). We conclude from these data that AST-004 cerebroprotective efficacy requires activation of the A3R.

### Neuroprotection mediated by the P2Y_1_R agonist MRS2365 is blocked by A3R antagonist

Our recent work suggested the neuroprotective efficacy of P2Y_1_R agonist MRS2365 (Zheng et al., 2010, 2013; Talley Watts et al., 2013), was mediated by the rapid production of an MRS2365 metabolite, AST-004 (Liston et al., 2020). To test the dependence of MRS2365 mediated neuroprotection on A3R agonism, we pretreated mice with the A3R antagonist MRS1523 as described above. As previously reported, MRS2365 treatments significantly reduced lesion volumes (14.31 +/− 1.81 mm^3^, *n* = 20) compared to untreated mice (23.74 +/− 2.17 mm^3^, *n* = 18) (Figures 3C,D). In mice treated with MRS1523, the average lesion volume reduction was (25.17 +/− 3.38, *n* = 12), not significantly different than control mice.

**FIGURE 4:**
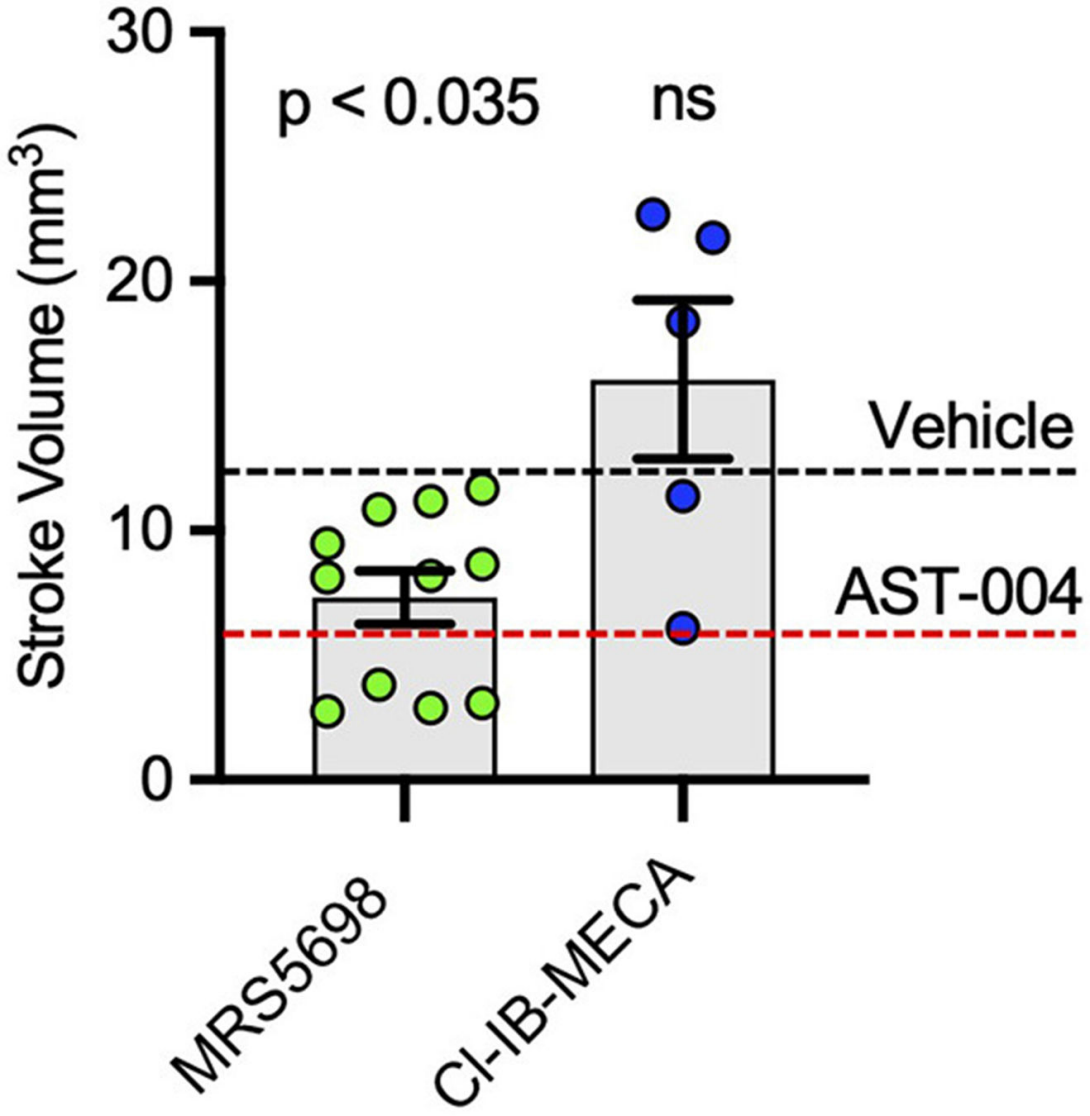
Cerebroprotective efficacy of A3R agonists MRS5698 and Cl-IB-MECA in photothrombosis-induced stroke infarctions. Average TTC-stained stroke volumes as labeled. Black dashed line shows mean stroke volume for untreated mice. Red dashed line shows mean stroke volume for AST-004 treated mice as presented in Figure 1B. Statistics relative to vehicle control in Figure 1B. Data were pooled from two experiments (*n* = number of mice per treatment) and plotted as mean +/− SEM.

We also tested the efficacy of the high affinity A3R agonists MRS5698 and Cl-IB-MECA. MRS5698 treatments (1.4 mg/kg) significantly reduced the mean lesion volume (7.33 +/− 1.07 mm^3^, *n* = 11) compared to untreated mice, although this reduction lesion size appeared smaller than that observed for AST-004 (Figure 4). However, in mice treated with the A3R agonist Cl-IB-MECA (0.19 mg/kg), the mean lesion size 24 h post-stroke (16.06 +/− 3.18, *n* = 5) trended higher than untreated mice (Figure 4). This may be due to the low blood barrier permeability of this compound or low unbound brain fraction available to interact with A3R. These data suggest that A3R agonism by itself, may not be sufficient for cerebroprotection as both MRS5698 and Cl-IB-MECA have substantially higher affinity for A3R than does AST-004, but this higher affinity does not translate to higher efficacy.

### AST-004 exerts its cerebroprotective effects through astrocyte energy metabolism

**FIGURE 5:**
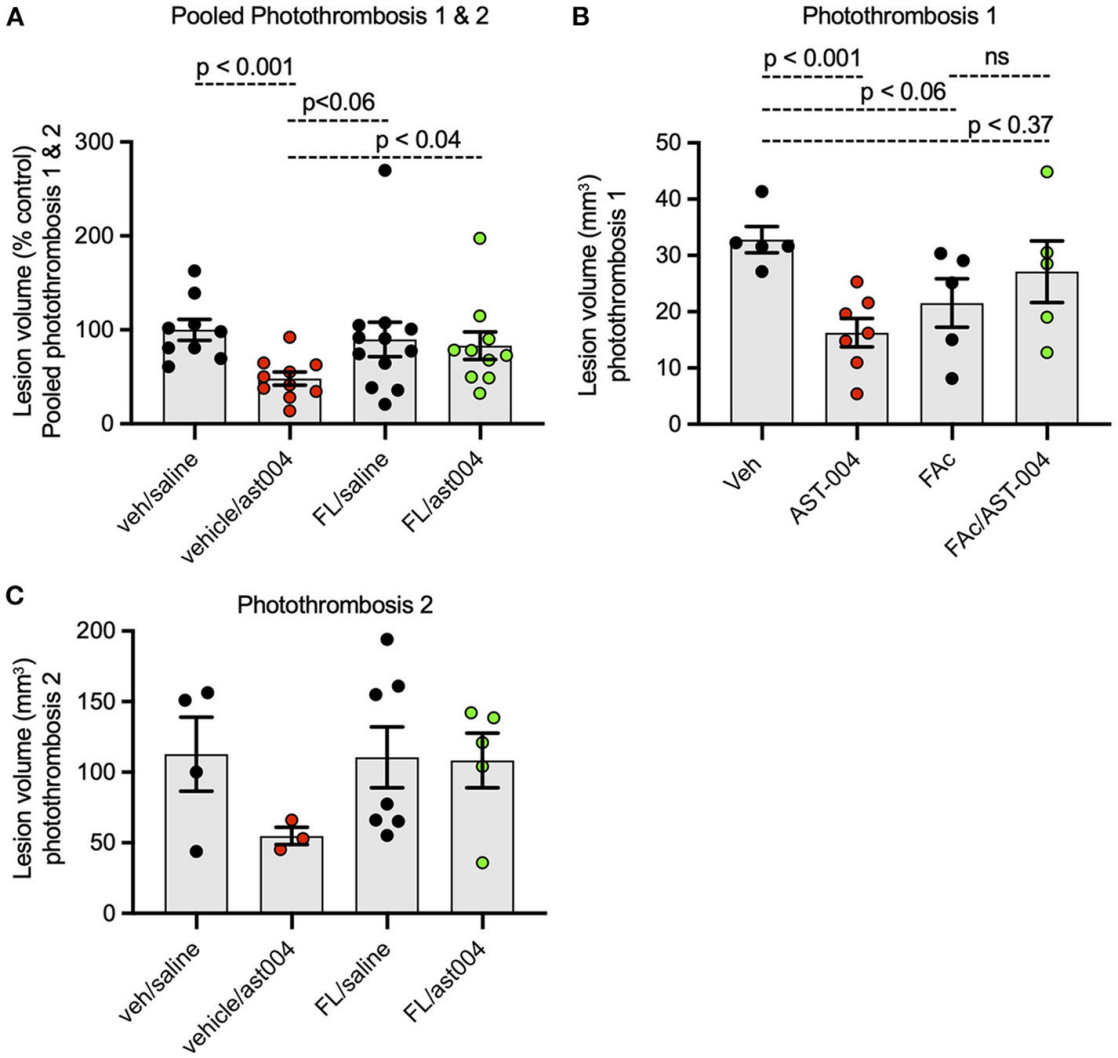
Fluoroacetate, an astrocyte specific mitochondrial toxin, blocks AST-004 neuroprotection. **(A)** Histogram plot of average TTC-stained stroke volumes in mice treated with either vehicle, AST-004, fluoroacetate (FAc) or fluoroacetate, and AST-004 (FAc/AST-004) expressed as a percentage of the average control volume. Data were pooled from mice receiving strokes using Photothrombosis methods #1 and #2. **(B)** Stroke volume data presented in **(A)** using only Photothrombosis method #1. **(C)** Stroke volume data presented in **(A)** using only Photothrombosis method #2.

Earlier studies by our group indicated MRS2365 cerebroprotection was the result of enhanced mitochondrial energy metabolism in astrocytes (Zheng et al., 2010, 2013). To test whether the cerebroprotection efficacy of AST-004, a MRS2365 metabolite, was similarly dependent on mitochondrial metabolism, we pre-treated mice with the astrocyte specific mitochondrial toxin, fluoroacetate (FAc, 0.004 mg/kg) (Fonnum et al., 1997). Consistent with the data presented above, treatment of mice with AST-004 alone significantly reduced the mean lesion volume to 48.09 +/− 6.98% (*n* = 10, *p* < 0.001) of control (100 +/− 11.01%, *n* = 9), 24 h post-stroke. In mice treated with FAc, however, AST-004 did not significantly reduce lesion sizes (83.08 +/− 14.66%, *n* = 10) (Figure 5A). Mice treated with vehicle and FAc exhibited an average lesion size of 89.75 +/− 18.33% (*n* = 12), not significantly different from control mice. We conclude from these data that the cerebroprotective efficacy of AST-004 is dependent on astrocyte mitochondrial metabolism.

### Photothrombotic stroke increases mRNA levels for *Adora3* and *Gfap* in the areas surrounding the lesion

**FIGURE 6:**
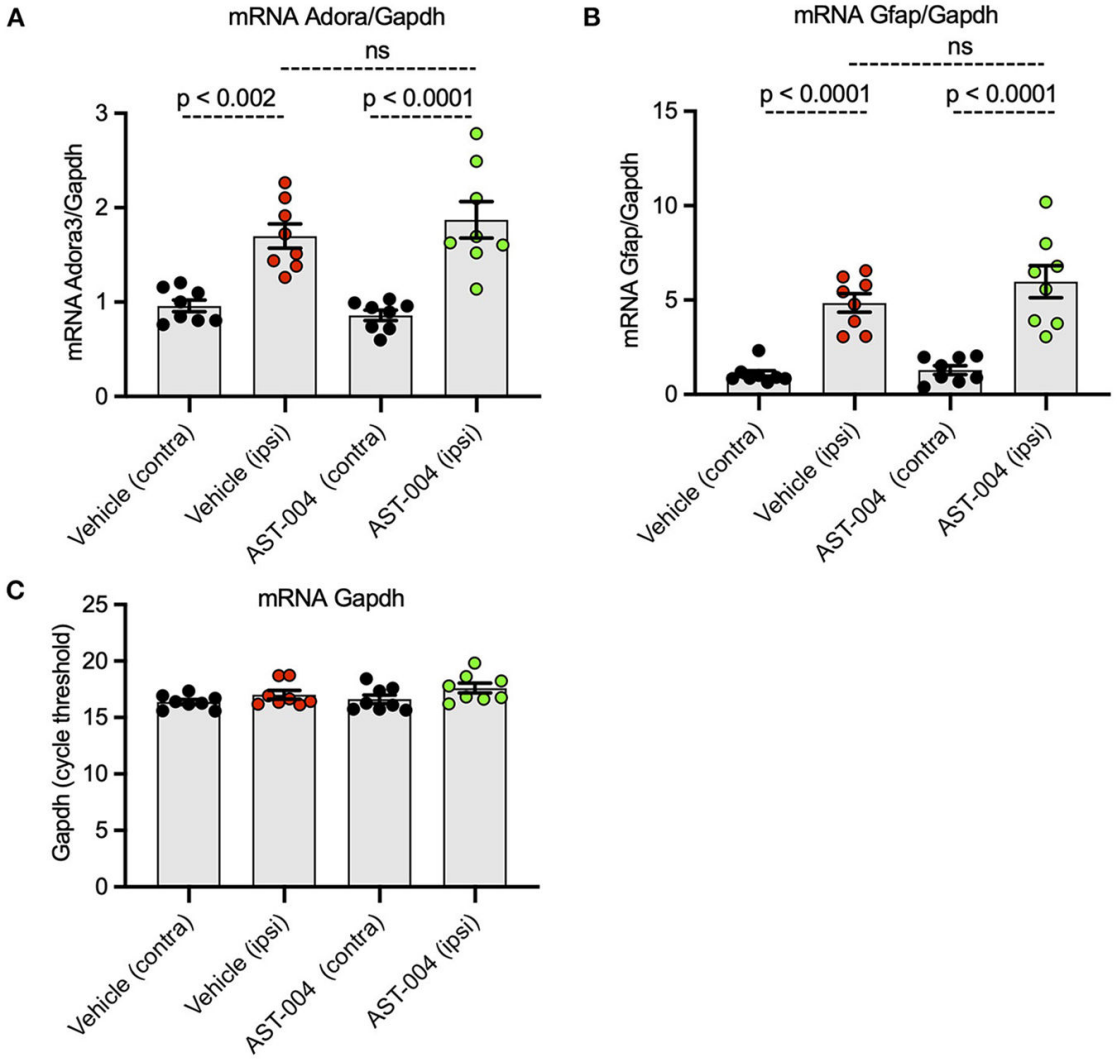
Ipsilateral mRNA levels of *Adora3* and *Gfap* are significantly increased 24 h post-stroke. **(A)** Relative quantity of *Adora3* mRNA in tissue surrounding photothrombotic lesion site. **(B)** Relative quantity of *Gfap* mRNA in tissue surrounding photothrombotic lesion site. Mice were sacrificed 24 h post-stroke, their brains removed, mRNA extracted and prepped for qRT-PCR as described in text. **(C)** Amplification plots for *Gapdh*, used as a control, in the tissues surrounding the photothrombotic lestions.

Numerous reports have indicated A3R expression is upregulated in the context of hypoxia and inflammation (Morschl et al., 2008; Godini et al., 2018; Torres et al., 2019). To determine if similar changes occur with our model of stroke, we isolated brain tissue surrounding the lesion 24 h post-stroke for qPCR measurements. We examined mRNA levels for both *adora3* and *gfap*, as a marker for astrogliosis, and compared the relative levels between ipsilateral and contralateral samples. We found a 70% increase in ipsilateral levels of *adora3* mRNA (1.70 +/− 0.13, *p* < 0.0001, *n* = 8) compared to contralateral brain tissue in untreated mice (0.96 +/− 0.06, *n* = 8) (Figure 6A). We also found ipsilateral levels of *gfap* mRNA (4.85 +/− 0.49, *p* < 0.0001, *n* = 8) increased nearly 5-fold relative to contralateral tissue (1.08+/− 0.19, *n* = 8), consistent with reactive astrogliosis occurring in non-treated injured mice (Figure 6B). We found similar changes in mRNA levels for both *adora3* and *gfap* in mice treated with AST-004. Adora3 mRNA levels increased to 1.87 +/− 0.19 on the ipsilateral side compared to 0.86 +/− 0.05 on the contralateral side (*p* < 0.0001, *n* = 8). Gfap mRNA levels increased to 5.98 +/− 0.85 ipsilateral compared to 1.30 +/− 0.23 on the contralateral side (*p* < 0.002, *n* = 8).

### AST-004 is cerebroprotective in rats after transient middle cerebral artery occlusion

**FIGURE 7:**
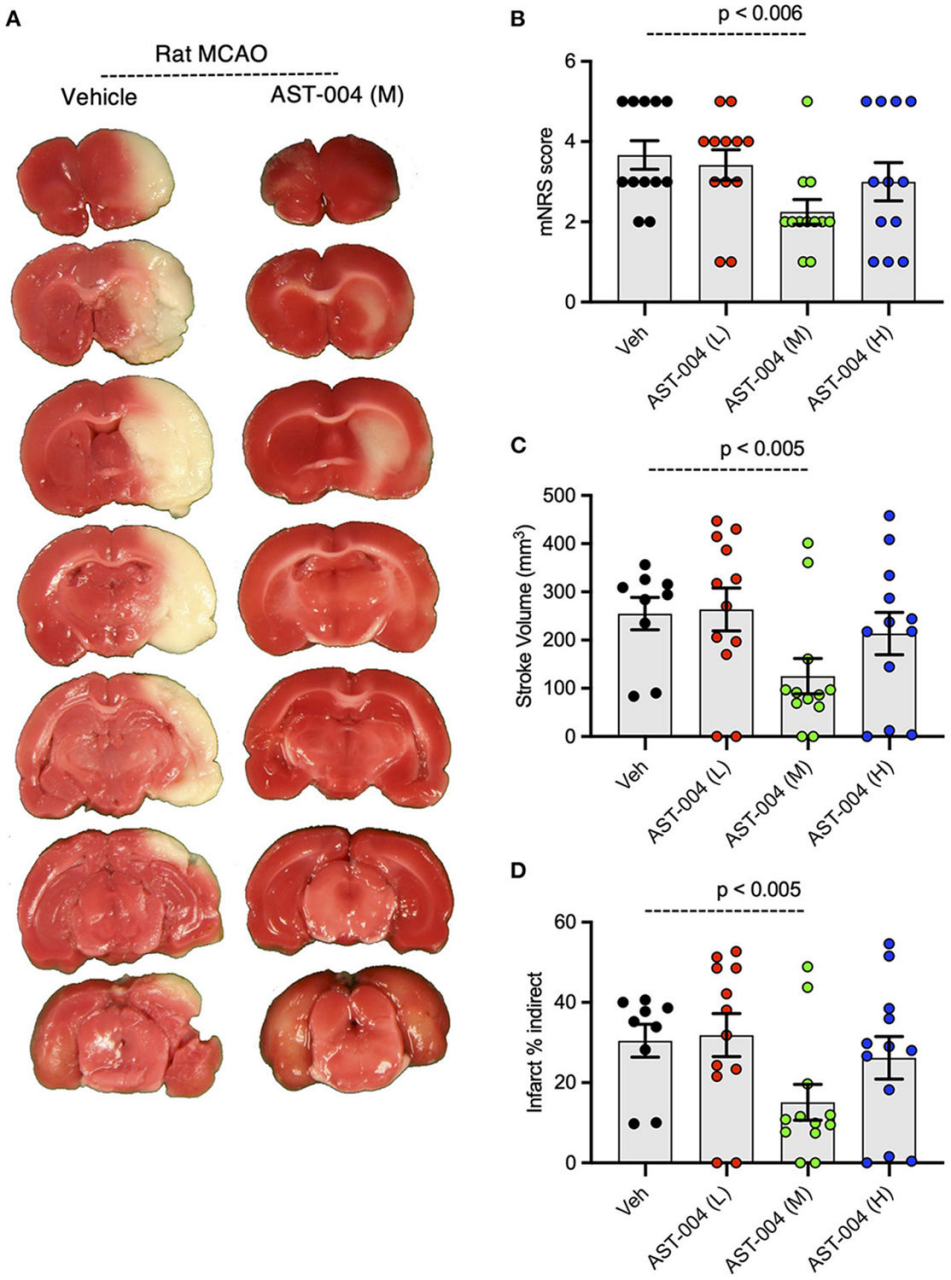
Dose-response of AST-004 treatments for MCAO stroke infarctions in rats. **(A)** Coronal sections of rat brains with MCAO infactions for vehicle (saline injected) and AST-004 (mid dose) treated mice. **(B)** Histogram of mNRS scores of animals at each dose of AST-004 tested. **(C)** Average TTC-stained stroke volumes in rats treated with either vehicle or AST-004 at the concentrations labeled: 0.04 mg/kg (L), 0.4 mg/kg (M), and 3 mg/kg (H). **(D)** Indirect measurement of lesion volume through comparative measurements of contralateral hemisphere at doses of AST-004 labeled. Data were collected from male mice and plotted as mean +/− SEM.

Given the efficacy of AST-004 in our photothrombotic mouse model of stroke, we also tested whether our A3R agonist was an effective treatment in a transient rat model of stroke. For these experiments, we induced stroke using middle cerebral artery occlusion (MCAO) for 90 min followed by reperfusion. AST-004 was intravenously injected at the start of reperfusion followed by constant rate infusions to maintain targeted concentrations through the course of the evaluation period. Three doses of AST-004, a full log difference from each other, were tested. Twenty-four hours post-stroke, we evaluated the functional activities of rats using the modified neurological rating scale (mNRS) (Longa et al., 1989). For this assay, no neurologic deficit is scored 0, a failure to extend the left forepaw is scored 1, circling to the left is scored 2, falling to the left is scored 3. Rats that do not walk spontaneously are scored 4 and rats that die are scored 5. For untreated rats, 83% scored between 3 and 5 with a mean mNRS of 3.67 +/− 0.36 (n = 12) (Figures 7A,B). For rats treated with the mid-dose of AST-004, only 25% scored between 3 and 5, resulting a significantly lower mean mNRS score of 2.25 +/− 0.30 (*n* = 12, *p* < 0.006). Average mNRS scores for the low-dose and high-dose of AST-004 were 3.42 +/− 0.38 (*n* = 12) and 3.00 +/− 0.48 (*n* = 12), respectively. Neither of these mNRS scores were significantly different from untreated rats. Immediately after mNRS scoring, brains were harvested, coronally sliced into 2 mm sections, then stained with TTC as described for mouse brains. The infarct volume for untreated rats, 24 h post-stroke, was 254.9 +/− 33.58 mm^3^ (*n* = 9) (Figures 7A,C). We found AST-004 treatments at the mid-dose (M) significantly (p < 0.005) reduced the mean stroke size to 125.3 +/− 36.75 mm^3^ (*n* = 12). The average lesion size in rats treated with the low-dose (L) of AST-004 was 263.8 +/− 44.53 mm^3^ (*n* = 12) and for rats treated with the high-dose of AST-004, the average lesion size was 213.8 +/− 43.90 mm^3^ (*n* = 12). Neither of these averages were significantly different from untreated rats. Essentially identical findings were observed when infarct volume measurements were replotted as a percentage of the uninjured hemisphere size (Figures 7A,D).

## Discussion

Our group previously demonstrated significant cerebroprotection after brain injuries using P2Y_1_R agonists MRS2365 and 2-MeSADP (Zheng et al., 2010, 2013; Talley Watts et al., 2013). Subsequent work showed these phosphorylated nucleotides were rapidly metabolized *in vivo* and that the active cerebroprotective compounds were likely the metabolites of MRS2365 and 2MeSADP, AST-004 and 2-methylthioadenosine, respectively (Liston et al., 2020). We confirmed AST-004 treatments were cerebroprotective after TBI in mice (Bozdemir et al., 2021) and tMCAO in non-human primates (Liston et al., 2022). Binding studies showed AST-004 was primarily an A3R agonist with some affinity for A1Rs (Liston et al., 2020).

Here, we validated the cerebroprotective efficacy of AST-004 following photothrombotic stroke in mice, a permanent model of ischemia, as well as following tMCAO in rats, a transient model of ischemia. Efficacy in both permanent and transient ischemia could be of significant clinical importance patients that achieve blood clot removal from either thrombectomy and/or thrombolysis, the current standard of care, and for patients who do not. We also found no sex-dependent effects of this AST-004. This is an important finding considering the often-reported differential effects drugs can have on men and women in clinical settings. Women are known to have a higher incidence of stroke, larger infarcts, and worse outcomes, which may be due, in part, to the fact women live longer than men (Girijala et al., 2017). However, when similarly treated, for example with tissue plasminogen activator (tPA), the difference in outcome measure is significantly reduced (Ahnstedt et al., 2016; Berglund et al., 2017). The higher incidence of strokes in women may also be affected by the loss of estrogen after menopause (Franconi et al., 2012; Pabbidi et al., 2018). Lesion sizes in ovariectomized rodent models are significantly larger, consistent with cerebroprotective effects of estrogens (Suzuki et al., 2009). In our experiments, the average lesion size in untreated female mice trended (*p* < 0.078) smaller than untreated male mice. Hence, it is possible our female mice benefited from endogenous actions of estrogen. Importantly, when normalized by the mean lesion size for vehicle treated mice, AST-004 significantly decreased lesion size in both females and males.

AST-004 is a lower affinity A1R/A3R agonist that exhibits significant efficacy as shown here and reported previously (Bozdemir et al., 2021; Liston et al., 2022). The cerebroprotective benefits of AST-004 were completely blocked by the A3R antagonist MRS1523, suggesting A1R agonism is not required. Moreover, MRS2365 cerebroprotection was completely blocked by the A3R antagonist, confirming MRS2365 served as a prodrug for AST-004 efficacy. Receptor binding models indicate significant efficacy is observed at estimated brain receptor occupancy levels as low as 5% in a non-human primate model of stroke (Liston et al., 2022). In light of these results, we tested AST-004 efficacy at doses an order of magnitude lower and higher than previously reported in mice (Bozdemir et al., 2021). All AST-004 concentrations were cerebroprotective against stroke, and the lower and higher doses were not significantly different from the mid-dose in mice. In rats, we observed a pronounce U-shaped dose response for both infarct size and the observed neurological deficits. Hormesis, or a “U-Shaped” biphasic dose-response has been observed with many CNS-active agents (Hammarberg et al., 2003; Calabrese, 2008). Importantly, no hormesis was observed in primate stroke efficacy studies, in which a clear AST-004 dose- and concentration-related effect was observed on inhibition of stroke lesion growth (Liston et al., 2022).

The observed hormesis in the rat stroke studies, not seen in either mouse or non-human primate studies under our experimental conditions, could be due to the dual affinities of AST-004 for A1Rs and A3Rs. Agonism of both adenosine receptor subtypes can be cerebroprotective in models of ischemic stroke (Cunha, 2001, 2005; Zamani et al., 2013; Tregub et al., 2014; Solino et al., 2018). However, A1R agonism has also been associated with cardiovascular effects including bradycardia and hypotension (Koeppen et al., 2009; Borea et al., 2018). Hypotension has been demonstrated to lead to higher stroke volumes and worse clinical outcomes in ischemic stroke as well as TBI. It is possible that a balance of AST-004 A1R and A3R agonism in rats results in significant efficacy, but at high doses, the peripheral cardiovascular effects of A1R agonism reduce this cerebroprotection. Again, this appears to be a rat-specific phenomenon, since we did not observe any evidence of hormesis or blood pressure effects over a broad dose range in a recent primate stroke efficacy study (Liston et al., 2022). More research is required to test the role of A1R agonism at higher doses in our stroke models.

Despite the significantly higher and more specific affinity of MRS5698 and Cl-IB-MECA for the A3R, those compounds had only similar or lower efficacy than AST-004 in the mouse photothrombotic stroke model. This may be due to the pharmacokinetic effects of the significant physicochemical differences between these compounds. AST-004 is a hydrophilic, polar small molecule whereas both MRS5698 and Cl-IB-MECA have substantially higher lipophilicity. These physicochemical differences lead to substantially higher plasma protein and brain tissue binding for MRS5698 (Tosh et al., 2015) and Cl-IB-MECA with correspondingly lower unbound fractions of compound required for distribution and receptor engagement compared to AST-004 (unpublished data). Thus, despite their high affinity for A3R, there are extremely low unbound fractions of these compounds in the brain for A3R agonism. In addition, previous data have suggested that both MRS5698 and Cl-IB-MECA may be excellent substrates for efflux transporters such as P-glycoprotein, potentially further limiting their distribution into the brain (Tosh et al., 2015; Abel et al., 2019). AST-004 is not a substrate for P-glycoprotein (unpublished data).

AST-004 cerebroprotection was blocked by the astrocyte specific mitochondrial toxin, fluoroaceteate. Fluoroacetate is preferentially transported into astrocytes by monocarboxylic acid transporter isoforms that are not present in neurons. Its toxic metabolite, fluorocitrate, inhibits the tricarboxylic acid cycle (Fonnum et al., 1997). These data are consistent with previous work showing cerebroprotection with the prodrug MRS2365 was dependent on astrocyte mitochondrial metabolism (Zheng et al., 2010, 2013). In addition, our group demonstrated cerebroprotection by these pro-drugs could be blocked by knocking out the astrocyte specific IP_3_R type 2 or by disrupting astrocyte specific mitochondrial function (Zheng et al., 2010, 2013). Together, these data suggest AST-004 acts by stimulating IP3-mediated Ca^2+^ release, leading to enhanced Ca^2+^ sensitive enzyme activity in astrocyte mitochondria. We note that A_3_Rs can effectively couple to either G_q/11_ or G_i/o_ subtypes of G proteins (Gilman, 1987; Abbracchio et al., 1995).

A3Rs are normally expressed at very low levels in the brain (Jacobson et al., 1993; Ji et al., 1994; Lopes et al., 2003; Gessi et al., 2008). Following photothrombotic stroke, we found a significant increase in brain A3R transcripts, which may aid long-term recovery after brain injury. The availability of additional A3Rs could minimize inherent problems with desensitization from either high levels of endogenous adenosine, which occurs after trauma (Bell et al., 1998) or in the presence of an exogenous A3R agonist. Regardless, it is clear mice null for A3Rs exhibit significantly worse outcomes after ischemic injury (Cheng et al., 1993; Fedorova et al., 2003; Chen et al., 2006). Interestingly, treatment of mice with AST-004 did not reduce the observed increases in either *adora3* or *gfap* mRNA levels by the 24 h timepoint post-stroke. Our group previously reported significant AST-004 mediated reductions in GFAP protein levels 3 days post-injury as well as reductions in *gfap* mRNA levels 7 days post-injury (Bozdemir et al., 2021). The data presented here suggest early cerebroprotective increases in mRNA immediately post-injury are not affected by AST-004. Rather, levels of *adora3* and *gfap* mRNA appear to subside only after AST-mediated healing occurs. Mechanistically, activation of A3Rs is known to inhibit proinflammatory cytokines (Tosh et al., 2014; Wahlman et al., 2018; Jacobson et al., 2020). Patients with autoimmune inflammatory diseases exhibit high expression of A_3_Rs in peripheral inflammatory cells and in blood mononuclear cells (Bar-Yehuda et al., 2007; Ochaion et al., 2009). A3Rs are overexpressed in the hypoxic core of tumors and have anti-cancer effects (Fishman et al., 2002; Madi et al., 2004). Protein and mRNA levels of A3Rs were also observed to increase after subarachnoid hemorrhage in rats (Li et al., 2020). It is unclear whether our energy-dependent mechanism of cerebroprotection is linked to these anti-inflammatory effects of A3R agonism. Production of reactive oxygen species (ROS) by mitochondria stimulate an inflammatory response (Fishman et al., 2012). Future studies are needed to test whether ROS production is reduced by AST-004 treatments. Independent, but complimentary mechanisms are also possible. Choi and co-workers reported A3R agonists reduced the lesion volume in rats after MCAO, which significantly decreased recruitment of inflammatory cells to the lesion site (Choi et al., 2011).

Overall, we have shown that AST-004 treatments provide significant cerebroprotection in two rodent models of stroke, including treatment of both transient and permanent occlusions. Pharmacological interventions indicate a dependence of this cerebroprotection on A3R agonism and mitochondrial metabolism in astrocytes. Together, these pre-clinical studies confirm the efficacy of AST-004 treatments after brain injuries and encourage the continued development of this new therapeutic in clinical trials.

## Supplementary Material

Supplementary File **SUPPLEMENTARY FIGURE 1** Overview of AST-004 study protocols in mice and rats. **(A)** Timeline of photothrombosis, injections and measurements in mouse protocol. **(B)** Timeline of MCAO, injections, and perfusions and measurements in rats.

## Data Availability

The original contributions presented in the study are included in the article/Supplementary material, further inquiries can be directed to the corresponding author.

## Abbreviations:

2MeS-ADP: 2-methylthio-adenosine-5′-diphosphate
A_1_R: Adenosine A_1_ Receptor
A_3_R: Adenosine A_3_ Receptor
AAALAC: Association for Assessment and Accreditation of Laboratory Animal Care, International
AIS: acute ischemic stroke
ANOVA: analysis of variance
AR: adenosine receptor
AST-004: (1R,2R,3S,4R,5S)-4-(6-amino-2-(methylthio)-9H-purin-9-yl)-1- (hydroxymethyl)bicyclo [3.1.0] hexane-2,3-diol
ATP: adenosine 5′-triphosphate
Ca^2+^: calcium ion
CO_2_: carbon dioxide
CCA: common carotid artery
Cl-IB-MECA: 2-Cl N^6^-(3-chloro or iodobenzyl)-adenosine-5′-N-methylcarboxamide
cm: centimeter
CNS: central nervous system
ddH_2_O: double deionized water
DEPC: Diethyl pyrocarbonate
DMSO: dimethyl sulfoxide
ECA: external carotid artery
FDA: food and drug administration
FAc: fluoroacetate
GAPDH: glyceraldehyde 3-phosphate dehydrogenase
GFAP: glial fibrillary acidic protein
IACUC: institutional animal care and use committee
ICA: internal carotid artery
IP: intraperitoneally
kg: kiligrams
MCAO: middle cerebral artery occlusion
mg: milligrams
ml: milliliter
mm: millimeter
mM: millimolar
MRS2365: (N)-methanocarba-2MeSADP
MRS5698: Molecular Recognition Section compound 5698
MRS1523: Molecular Recognition Section compound 1523
mNRS: modified neurological rating scale
nM: nanomolar
NIH: National Institutes of Health
NHP: non-human primates
P2Y_1_R: P2Y type 1 receptor
PBS: Phosphate buffered saline
PFA: paraformaldehyde
PK: pharmacokinetics
PE: Polyethylene
qRT-PCR: quantitative real-time polymerase chain reaction
ROS: reactive oxygen species
Rpm: revolutions per minute
SDS: sodium dodecyl sulfate
tPA: tissue plasminogen factor
TTC: 2,3,5-Triphenyltetrazolium chloride
RNA: ribonucleic acid
S.E.M.: standard error of the mean
