# Corrigendum: Adenosine A1R/A3R agonist AST-004 reduces brain infarction in mouse and rat models of acute ischemic stroke

**DOI:** 10.3389/fstro.2024.1371734

**Published:** 2024-01-29

**Authors:** Elizabeth S. Fisher, Yanan Chen, Mikaela M. Sifuentes, Jeremy J. Stubblefield, Damian Lozano, Deborah M. Holstein, JingMei Ren, Matthew Davenport, Nicholas DeRosa, Tsung-pei Chen, Gerard Nickel, Theodore E. Liston, James D. Lechleiter

**Affiliations:** ^1^Department of Cell Systems and Anatomy, University of Texas Health at San Antonio, San Antonio, TX, United States; ^2^NeuroVasc Preclinical Services, Inc., Lexington, MA, United States; ^3^Astrocyte Pharmaceuticals Inc., Cambridge, MA, United States

**Keywords:** *Adora3*, stroke treatment, mitochondrial metabolism, ATP, astrocytes

In the published article, there was an error in the Funding statement. The funding source for coauthor Jeremy J. Stubblefield was not included. Original text: This study received funding from Astrocyte Pharmaceuticals, Inc. The correct Funding statement appears below.

## Funding

This study received funding from Astrocyte Pharmaceuticals, Inc. JS was supported by NIH IRACDA training grant K12 GM111726.

The authors apologize for this error and state that this does not change the scientific conclusions of the article in any way. The original article has been updated.

