# Supplementary figures and images for: Adenosine A1R/A3R agonist AST-004 reduces brain infarction in mouse and rat models of acute ischemic stroke
Source: Front Stroke. Author manuscript; Available in PMC 2024 Feb 12. (PMC10861240; doi:10.3389/fstro.2022.1010928)

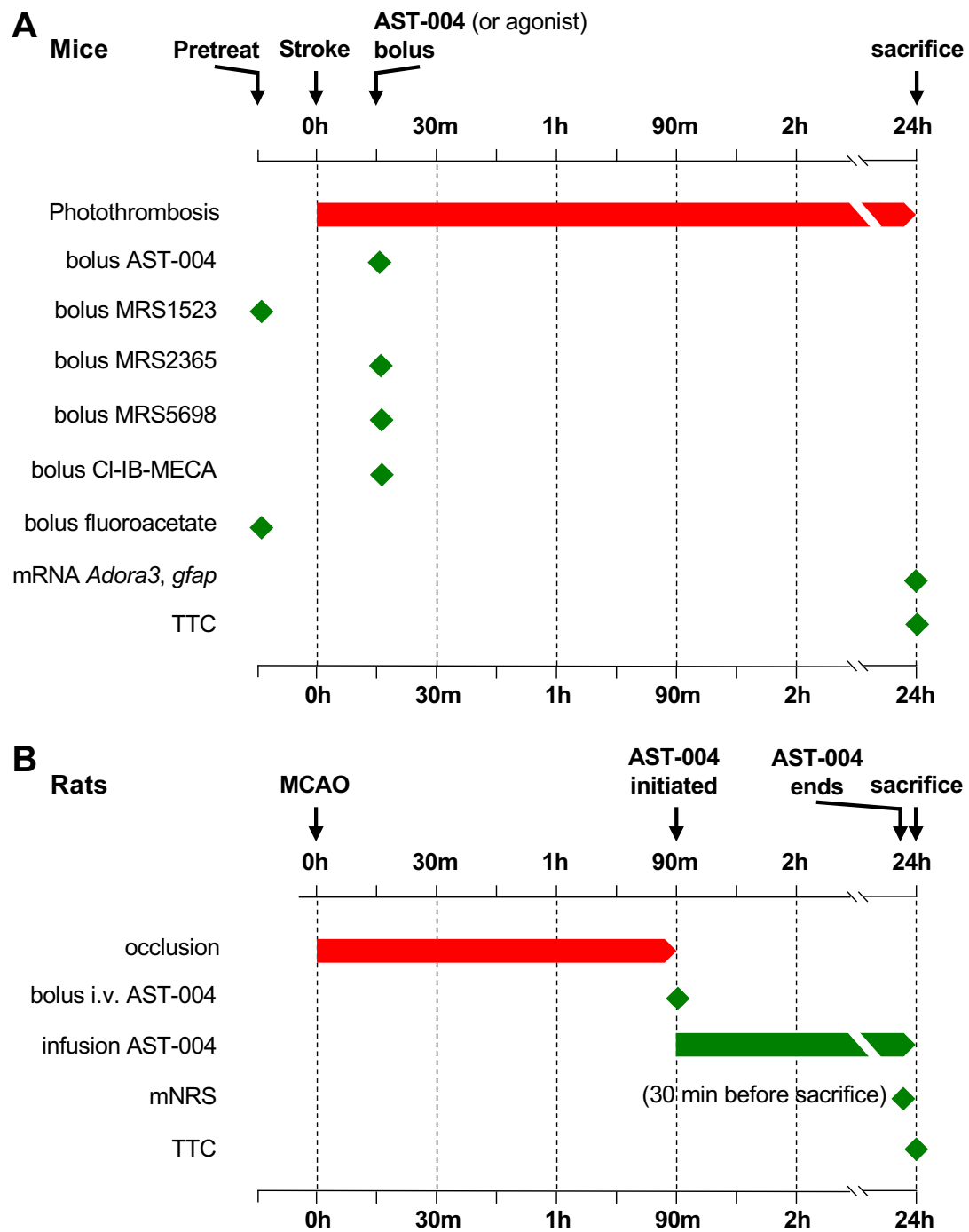

Supplementary Figure 1

Supplement: Supplementary File — SUPPLEMENTARY FIGURE 1 Overview of AST-004 study protocols in mice and rats. (A) Timeline of photothrombosis, injections and measurements in mouse protocol. (B) Timeline of MCAO, injections, and perfusions and measurements in rats. [file NIHMS1961017-supplement-Supplementary_File.pdf]
